# History of Balkan Medical Journal: Road to High-Impact Journal

**DOI:** 10.4274/balkanmedj.2018.1.0001

**Published:** 2018-01-20

**Authors:** Zafer Koçak, Ahmet Ulugöl

**Affiliations:** 1 Department of Radiation Oncology, Trakya University School of Medicine, Edirne, Turkey; 2 Department of Medical Pharmacology, Trakya University School of Medicine, Edirne, Turkey

The recent dramatic development of the Balkan Medical Journal (1), especially after 2005, led us to share the history of our journal with our readers and authors. Another reason for writing this editorial is to document the story of how the Balkan Medical Journal has gained respect in the Balkans and in the world.

## 1979-1980: Launching the Journal and Publishing the First Two Volumes

**Figure 1:**
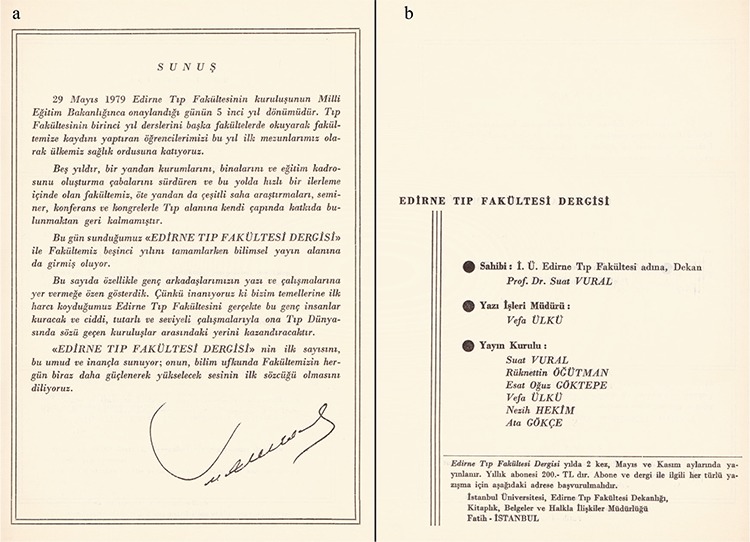
During the presentation of the first issue in May 1979, Professor Dr. Suat Vural, the owner of the journal and the dean of the medical faculty, launched a journal under the title of “Edirne Tıp Dergisi” (Edirne Medical Journal) in the field of general medicine, which he hoped would be the scientific voice of Edirne Medical Faculty (a), The founding editorial board (b).

The Balkan Medical Journal had its beginning under the title of “Edirne Tıp Dergisi” (Edirne Medical Journal) in May 1979 when Edirne Medical Faculty was a branch of İstanbul University in Cerrahpaşa Medical Faculty. In the summer of that year, the school gave its first graduates. During the presentation of the first issue, Professor Dr. Suat Vural, the owner of the journal and the dean of the medical faculty, launched a journal in the field of general medicine, which he hoped would be the scientific voice of Edirne Medical Faculty. Figure 1 shows the founding editorial board and the opening editorial.

The first two volumes were published in Istanbul. After movement from İstanbul to Edirne in 1982, Edirne Medical Faculty received a new name, “Trakya University School of Medicine.” Accordingly, “Edirne Tıp Dergisi” had its name changed to “Trakya Üniversitesi Tıp Fakültesi Dergisi” (Medical Journal of Trakya University). Interestingly, the first two volumes were published by three different editors (Vefa Ülkü, Zeynep Erdal, and Meliha Özkay); unlike today’s publishing conception, they were working more as an executive secretary than as an Editor-in-Chief.

## 1986-2005: Tough Times to Get Articles from Outside of Trakya University

**Figure 2:**
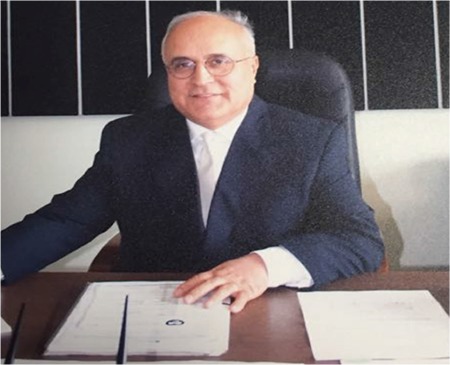
Professor Sebahattin Çobanoğlu was the Editor-in-Chief between 1986 and 1996. In the history of the journal, he was the longest serving editor.

After publishing the second volume in 1980, the publication of the journal was interrupted for 5 years during the foundation period of Trakya University in Edirne. Beginning from the third volume in 1986, the journal resumed its publication life under the patronage of Associate Professor Çobanoğlu (Figure 2). He continued this task for 10 years.

**Figure 3:**
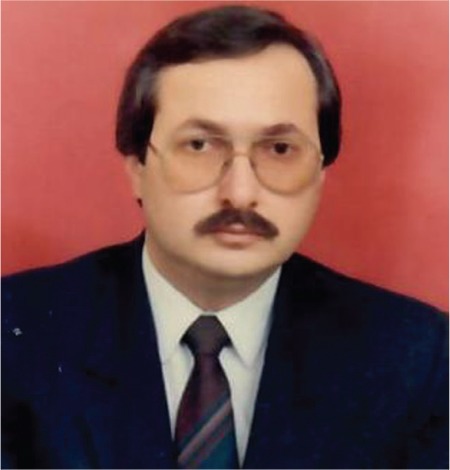
In 1996, Professor Ahmet Karasalihoğlu was appointed as the Editor-in-Chief and served until 2002. During his term, the journal started to be indexed in a database (Turkish Medical Index) for the first time. This was the presage of the steps that would be taken later.

In 1996, Professor Karasalihoğlu was appointed as the Editor-in-Chief and served until 2002 (Figure 3). During his 6-year term, the journal was accepted to be indexed in the Turkish Medical Index for the first time in 2000. Perhaps this was the presage of the steps that would be taken later.

**Figure 4:**
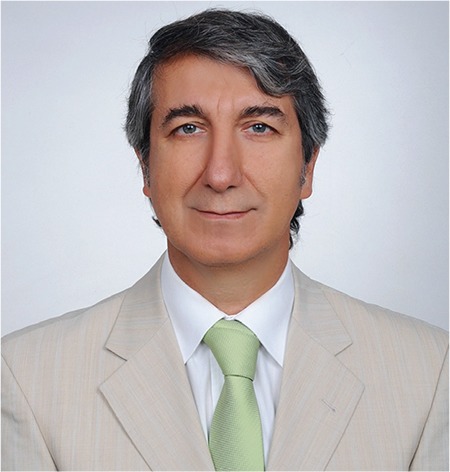
Professor Erol Yalnız was the Editor-in-Chief between 2002 and 2005. In 2003, the journal was indexed in an international database (Index Copernicus) for the first time.

In 2002, Professor Yalnız was appointed as a new Editor-in-Chief of the journal (Figure 4). Only a year later, he announced that Index Copernicus was the first international database where the journal was indexed. Despite this great news of those years, the primary source of submission was still Trakya University School of Medicine in Edirne (Table 1).

## 2005 and After: A respected Journal in the Balkans and in the World

**Figure 5:**
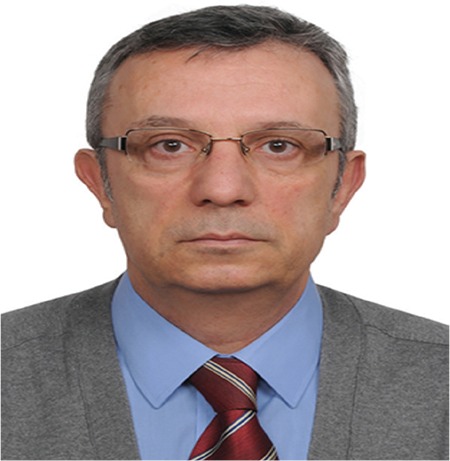
Professor Ahmet Ulugöl took over the task in the first quarter of 2005 and served until the end of 2008. The first web site of the journal and the use of online submission took place during Professor Ulugöl’s time. Under his leadership, the most important milestone for the journal has been realized and the journal started to be indexed in Science Citation Index Expanded.

Professor Yalnız resigned in the first quarter of 2005, and Professor Ulugöl took over the task (Figure 5). Professor Ulugöl started his task with a change in the Editorial Board and establishment of an international advisory board. In his first editorial, he stressed the importance of rapid peer-review and timeliness publication and set his sights on coverage of the journal in Index Medicus and Science Citation Index-Expanded. Commencing with his period, the journal also started to publish review articles only by invitation.

In 2005, with great efforts of Professor Ulugöl and the Associate Editors, the journal was covered by Ulrich’s Periodicals Directory, Oxford University Libraries, JournalSeek, and DOAJ. In 2006, the editorial team began to use the online submission and peer-review systems, which simplified and accelerated the publication process. These changes were important steps for the future of the Journal. Consequently, the number of submissions from outside of Trakya University began to increase steadily, reaching two-thirds of the total in 2007 (Table 2). At the same time, good news about indexing continued to come up, and the journal began to be indexed in Free Medical Journals, Chemical Abstracts, and EBSCO.

Three years after his leadership, some goals of Professor Ulugöl took place as it was mentioned in his first editorial. Certainly, the first important milestone for our journal was the inclusion into SCI-Expanded in 2008, which led to a significant increase in the number of submissions (Table 2). This striking increase in the number of submissions was partially due to the new criteria of the Higher Education Council in Turkey for applying for academic promotion. One of the criteria was to publish an original article in journals indexed in SCI-Expanded. Professor Ulugöl’s period lasted three and a half years, and he left his post as an Editor at the end of 2008.

**Figure 6:**
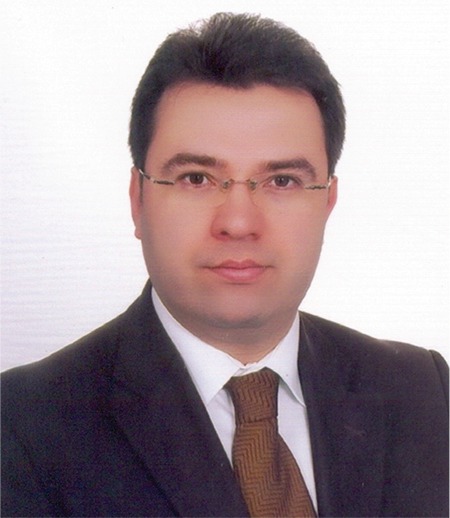
Professor Levent Öztürk, as an Editor-in-Chief, served between 2009 and 2012. On the way to becoming an international journal, some of the biggest decisions were taken during his term, i.e., changing the title of the journal and accepting articles only in English. Other achievements during his period were to get the DOI number for articles and to use a plagiarism software to raise ethical standards.

After Professor Ulugöl’s resignation, Associate Professor Öztürk was appointed as the Editor-in-Chief (Figure 6).

In fact, there is nothing wrong in calling his term as an era of several breaking changes. He immediately faced two challenges, i.e., increased number of submissions and financial problems. Beginning from January 2009, the editorial team decided to publish the journal quarterly and charge submission fee from authors.

In 2010, Professor Öztürk was informed that the journal was accepted to be indexed in Scopus and Embase. This was a great news for us as Scopus is a citation database. In the same year, we started to get DOI number for journal articles. A few months after DOI’s application, we began using a plagiarism software to raise ethical standards (2). On the other hand, probably due to submission fee, we observed a dramatic decline in the number submissions in 2010 and 2011.

On the way to becoming an international journal, some of the biggest decisions were taken in 2011. Changing the title of the journal was one of the boldest actions; the other critical decision was to accept articles only in English. This decision-making process was very difficult and extensive discussions were made at editorial meetings. The editorial board of the journal at that time believed that the title of an ideal journal should be short, clear, unique, indicative of the coverage of the publication, and easily remembered. Besides this, the title at that time was “Medical Journal of Trakya University” and it was found to be too local for the journal covered by international indexes. Thus, the new journal title, “Balkan Medical Journal,” was announced by Professor Öztürk and started to be used from volume 28 onward (Table 3).

In 2012, the last sentence of the farewell letter by Professor Öztürk was as follows “Your contributions are essential for the Balkan Medical Journal to help it achieve its goals which basically include contributing to medical sciences and healthcare in the Balkan area through increased scientific productivity and continuous medical education” (3).

**Figure 7:**
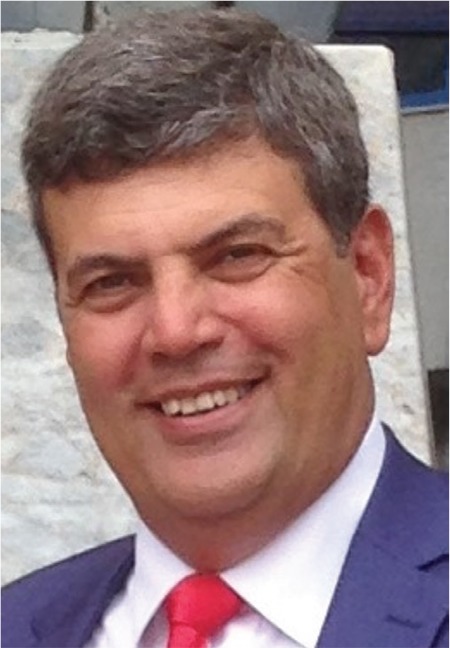
Professor Cem Uzun’s term began in 2012 and lasted until the fourth quarter of 2016. He made great efforts to establish international standards of publication ethics for the journal and close cooperation with the Balkan countries. The journal was started to be indexed in PubMed/Central in 2013. Other accomplishments during his period were membership in international organizations and shaping the journal’s training mission.

In the third quarter of 2012, Professor Uzun’s term started (Figure 7). In his period, the policy on cooperation with the Balkan countries was remarkable. One of the first attempts he made was to enhance the Editorial Board with Balkan scientists. His close links with scientists in the Balkan countries have increased the regional reputation of the journal. Some achievements during his editorship were updated instructions and editorial policy, acquisition of the ScholarOne Manuscripts peer-review system, and English editing service (4).

Professor Uzun has made great efforts to establish international standards of publication ethics for the journal. As a result of this, the journal became a member of important international organizations, including the Committee on Publication Ethics (COPE), the International Council of Medical Journal Editors (ICMJE), the World Association of Medical Editors (WAME), the Council of Science Editors (CSE), and the European Association of Science Editors (EASE). With those memberships, the journal became committed to following their instructions (5).

In 2013, we were informed that the journal was accepted to be indexed in PubMed/Central. This was very good news for the visibility and prestige of the journal. At the end of the same year, we decided to do something more for the journal’s prestige, and beginning from 2014, the submission fee was abandoned. These developments led to a dramatic increase in the number of submissions in 2015. Starting from 2016, the increase in the number of articles forced us to publish the journal bimonthly.

**Figure 8:**
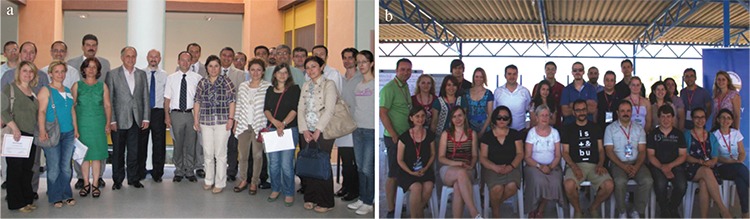
The Balkan Medical Journal has a national and an international training mission. Training of authors program, attended by all editors of the Balkan Medical Journal, was held for the first time in the Central Library Halls of Trakya University on May 30, 2013, as a national scale of the training mission (a), In 2014, the 11th session of the International Scientific Summer School was organized in Edirne under the patronage of the Balkan Medical Journal with the support of Trakya University as an international scale of the training mission (b).

Another characteristic of Professor Uzun’s period was the educational task of the journal. As read in Professor İnan’s editorial “In addition to independent, impartial and active sharing of scientific information with the whole world, the mission of the Balkan Medical Journal is to train the actors taking part in this process” (6). There are two scales of the training mission of the journal, national and international. In the national scale, the Editorial Board provides training on project design to researchers in the Thrace region (Figure 8a). In the international scale, the training activity is carried out in cooperation with the International Scientific Summer School (ISSS). The Balkan Medical Journal has been a member of the ISSS initiative since 2010. In 2014, the 11th session of the ISSS was organized in Edirne under the patronage of the Balkan Medical Journal with the support of Trakya University (Figure 8b) (7).

## Present and Future

**Figure 9:**
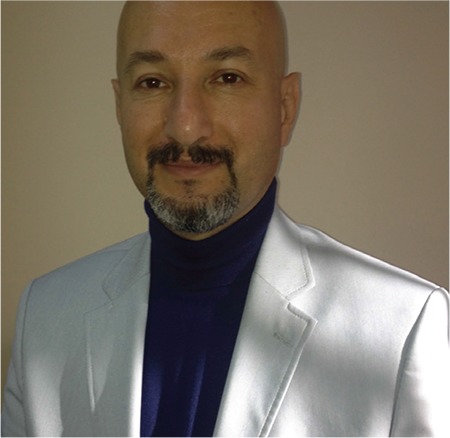
Professor Zafer Koçak was appointed as the Editor-in-Chief in the last quarter of 2016. For the first time in the journal’s history, an editorial independence agreement was signed between the Editor and the journal owner. In November 2016, the journal was accepted to be indexed in PubMed/MedLine. Within a year, the journal had a professional website and the articles and content of the journal were visible on social media.

In the fall of 2016, Professor Koçak handed over the editorial duty (Figure 9). For the first time in the journal’s history, an editorial independence agreement was signed between the Editor and the journal owner. This step was carried out in the hopes of starting a tradition. However, during the first days of their new appointment, the editorial team faced a publishing house change. In the meantime, in November 2016, we were informed that the journal was accepted to be indexed in PubMed/MedLine. Thus, one more of our goals was realized.

**Figure 10:**
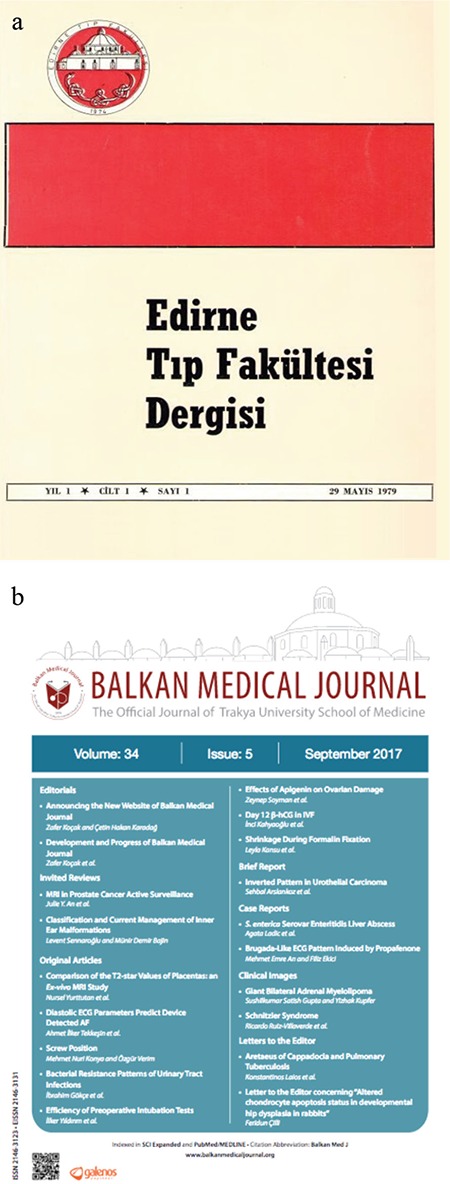
The Balkan Medical Journal had its beginning under the title of “Edirne Tıp Dergisi” (Edirne Medical Journal) in May 1979 (a), In 2011, the title of the journal was changed to “Balkan Medical Journal,” and the current cover of the journal began to be used from the third issue of 2017 onward (b).

Professor Koçak always believed that institutions must have a memory. Sultan Bayezid II complex of Edirne, founded in 1488, is the first example of a centrally planned medical center and is considered to be the forerunner of modern hospitals (8). Thus, the roots of medical education in the city date back to the foundation day of the school in Bayezid II complex. Starting from the third issue of 2017, Professor Koçak announced the new cover of the journal with the words “Balkan Medical Journal, symbolizing its historical heritage with the silhouette of Bayezid II Şifahanesi on its new logo and name during this new period, constitutes a visual memory of the transition of tradition to the future by a bold and innovative interpretation of tiling colors” (9). Figure 10 shows the first and the current covers of the journal.

One of the first things Professor Koçak did was to restructure the Editorial Board with the establishment of the Deputy Editors, the Editor-at-Large, and the Honorary Editor. Another innovation was the inclusion of brief reports and special section articles as new types of articles to be published. In the last quarter of 2017, Professor Koçak and Professor Karadağ (Web Editor) announced a new and improved website that will provide better service to readers, reviewers, and authors (10). Undoubtedly, this dynamic website will contribute to the visibility and recognition of the journal. Soon after, the articles and content of the journal became visible on social media.

The Balkan Medical Journal has now completed 38 years of publication and is well recognized by the international health community. By doing the right thing, especially applying high ethical standards, the Balkan Medical Journal now adheres to the internationally accepted criteria of modern medical journalism and has taken the well-deserved place in the Balkan region and in the world (11). Table 4 summarizes our struggle over the last 13 years on the way to its aim of becoming a high impact journal.

## Figures and Tables

**Table 1:**
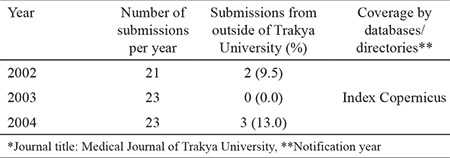
The number of submissions and the coverage by databases/directories between 2002 and 2004*

**Table 2:**
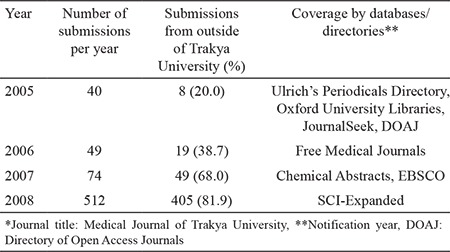
The number of submissions and coverage by databases/directories between 2005 and 2008*

**Table 3:**
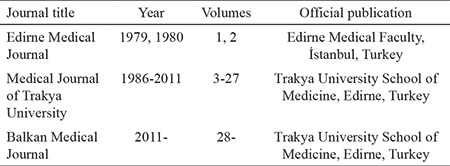
Publication titles, years, volumes and locations of Balkan Medical Journal

**Table 4:**
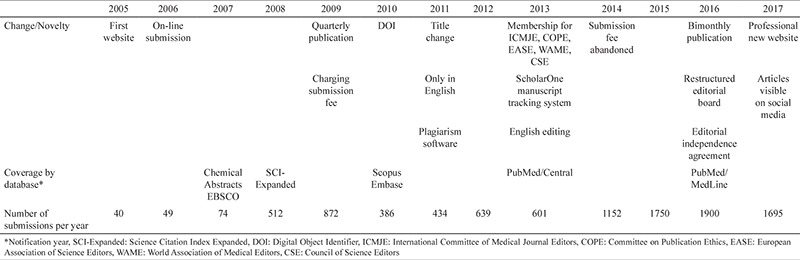
The number of submissions per year and important milestones achieved between 2005 and 2017
